# Maternal Vertical Transmission Provides a Mitochondrial Basis for ROP Susceptibility

**DOI:** 10.1167/iovs.67.12.78

**Published:** 2026-10-30

**Authors:** Kevin Fung, George Hoppe, Andrew Benos, Demiana Hanna, Ke Jiang, Jonathan E. Sears

**Affiliations:** 1 Ophthalmic Research, Cole Eye Institute, Cleveland Clinic, Cleveland, Ohio, United States; 2 Metabolomics & Cardiovascular Sciences, Cleveland Clinic Research, Cleveland Clinic, Cleveland, Ohio, United States; 3 Illinois Eye and Ear Infirmary, Department of Ophthalmology and Visual Sciences, University of Illinois Chicago, Chicago, Illinois, United States

**Keywords:** retinopathy of prematurity (ROP), mitochondria, oxidative phosphorylation, A/J mouse, glutamine (Gln), *Hmox1*

## Abstract

**Purpose:**

The purpose of this study was to link strain-based susceptibility to mitochondria.

**Methods:**

Crosses between susceptible and protected strains of mice were analyzed for oxygen-induced retinopathy (OIR). Oxygen consumption and extracellular acidification of retina explants from individual strains were quantified. Expression and protein concentrations of key metabolic proteins were measured. Isotopic based metabolomics was used to measure transformations of glycolytic carbon.

**Results:**

A/J mice were protected from OIR compared to C57Bl/6 mice, having a 5-fold reduction in the ratio of vaso-obliteration (*P* = 1*10^−5^) and a 20-fold reduction in neovascularization (*P* = 0.002). Mapping among A/J, Balb/C, and C57Bl/6 mice showed a maternal inheritance pattern of protection that implicated mitochondria. A/J and C57Bl/6 mitochondrial functions showed a reduction in the basal respiration rates of A/J ex vivo retina (*P* = 4*10^−9^) and a two-fold reduction in the carboxyethyl pyrrole oxidative stress marker of A/J mice retinas with OIR (*P* = 0.007) compared with C57Bl/6 mice. Placed in hyperoxia, A/J mice retina demonstrated reduced levels of respiratory proteins, as well as expression of subunit IVi1 of cytochrome c oxidase in comparison to C57Bl/6 (*P* = 3*10^−6^). To support the hypothesis that a reduction in oxidative phosphorylation was protective, C57Bl/6 mice were injected with the mitochondrial uncoupler 2,4 dinitrophenylhydrazine (DNP) during the hyperoxia phase of OIR; DNP created reduced levels of vaso-obliteration (*P* = 1.5*10^−5^) and neovascularization (*P* = 8*10^−6^). Isotopic metabolomics demonstrated that A/J aortic endothelium increased glutamine (Gln) synthesis from glycolytic carbon in comparison to C57Bl/6 with upregulation of *Hmox1* expression.

**Conclusions:**

Strain-specific susceptibility or protection may involve vertical maternal transmission.

Retinopathy of prematurity (ROP) is a leading cause of infant blindness, accounting for 100,000 new cases per year worldwide.^1^ The pathogenesis of ROP has been definitively linked to hyperoxia through preclinical rodent models and oxygen trials in humans.^2^^–^^4^ These studies have demonstrated that oxygen supplementation is necessary to reduce morbidity in premature infants, but also that hyperoxia is unfortunately causative to not only ROP but bronchopulmonary dysplasia and demyelination of the central nervous system.^5^ The causal relationship of oxygen is further confirmed by studies of the hypoxia-inducible factor, a pleiotropic transcription factor that regulates metabolic, erythropoietic, and cytoprotective mechanisms in response to varied oxygen tension.^6^^–^^8^ Two factors, low birth weight and early gestational age at birth, are risk factors contributing to the severity of ROP and correlate to the oxygen-based pathogenesis of ROP because they are associated with increased avascular retina, the substrate of ROP, at birth and after neonatal hyperoxia.^9^

Despite the enormous base of knowledge generated by basic and clinical research about ROP, including pathological and developmental angiogenesis in general, the role of genetic susceptibility to oxygen-induced retinopathy (IOR) is incompletely understood.^10^ For example, a cohort of patients were found to have severe ROP in conjunction with mutations in the *wnt* pathway.^11^ But outside of these systemic mutations perhaps inducing premature birth, these rare instances might be the coincidence of prematurity with these gene defects. Genome-wide association studies and whole exome sequencing comparing similar cohorts of infants with disparate ocular outcomes showed SNP *GLI3 rs2058019* (glioblastoma-related Zn finger protein) reaching genome-wide significance (*P* = 4.9*10^−9^) within a multiethnic cohort in the former study,^12^ whereas the latter investigation revealed no specific gene association but rather two pathway associations, vitamin C metabolism and smooth endoplasmic reticulum.^13^

However possible that these pathways and genes participate in susceptibility to ROP, it is well known that in rodents, the standard OIR model creates a robust disease phenotype in certain strains of mice, such as C57Bl/6, but not so in strains of mice with an albino background, such as BALB/c.^14^ In addition, retinal capillaries in BALB/c mice are more resistant to injury from ischemia/reperfusion experiments compared with C57Bl/6 mice.^15^ We and others have definitively demonstrated the role of metabolism in protection against OIR.^16^^–^^19^ Pathologic angiogenesis in the OIR model has been linked to a decrease in mitochondrial function in the proliferative phase 2 of ROP.^16^ In the latter study, a decrease in oxygen consumption occurred in relative hypoxia. In this investigation, we hypothesized that these strain-specific differences might harbor not genomic patterns of inheritance but rather mitochondrial gene differences. We found that the A/J mouse has near complete resistance to oxygen toxicity, demonstrating less vaso-obliteration during hyperoxia and complete revascularization by postnatal day 17 (P17), and that this protection followed maternal vertical inheritance patterns. Retina of protected strains used less oxygen during hyperoxia and overall had reduced protein levels of Complex I, II, III, and V in company with oxygen-induced dysregulation of a key subunit of Complex IV.

## Methods

### Animals

All experimental procedures involving live animals were approved by the Cleveland Clinic Institutional Animal Care and Use Committee (protocol no. 2904) in accordance with the ARVO statement for humane use of animals for research. Wild-type C57BL/6 and A/J mice were supplied by Jackson Laboratory.

### Oxygen Induced Retinopathy and Retinal Vasculature Analysis

OIR as an animal model of ROP used in this study is based on a well-established protocol by Smith et al.^13^ Pharmacological treatment of OIR with 20 mg/kg DNP was performed with daily intraperitoneal injections from P6 to P11. OIR-associated vascular pathology was assessed by computer-assisted quantification of retinal capillary loss and neovascular tufts in isolectin-stained, whole-retinal flat mounts using software developed by Xiao et al. [PMID: 29263301].

### Mouse Aortic Endothelial Cell Culture and Primary Harvest

Harvesting of mouse aortic endothelial cells was followed according to the protocol detailed by Wang et al.^14^ Briefly, the thoracic aorta is cleanly dissected from the mouse body and cut into 1 mm rings. The rings are seeded onto Matrigel and cultured with endothelial cell growth medium for 4 days. The rings are removed on day 4 to prevent the migration of other cell types other than endothelial cells. The cells were passaged at least five times before use in experiments. Primary cell cultures were created for the A/J and C57Bl/6 mouse strains.

### Choroidal Sprout Culture

To create the ex vivo choroid sprouting model, a protocol was followed as described by Shao et al.^15^ Briefly, eyes were harvested from A/J and C57Bl/6 pups at P10 of age, and their choroid tissue was dissected and cut into approximately 1 mm by 1 mm pieces. Then, 20 µL of Matrigel was used to coat the bottom of a 24-well plate, and a piece of choroid was seeded into this Matrigel. The cells were incubated at 37°C with DMEM media containing 20% fetal bovine serum (FBS) and 15 mg/mL ECGS. To assess survival of choroid sprouts in hyperoxia, sprouts were allowed to grow for 7 days before being placed in a 75% oxygen cell incubator for 5 days. Contrast photographs of each individual explant were taken daily using the Leica THUNDER Imaging system, and the areas of sprouting were quantified with ImageJ software (National Institute of Health).

### Western Blot Analysis

Mouse pups were euthanized by lethal injection of ketamine/xylazine, and their retinas were dissected and snap frozen in liquid nitrogen. Samples were homogenized in RIPA + protease inhibitor cocktail Complete (Roche) using 1 × 10 quick pulses of sonication. Protein extracts were cleared by 20,000 × g centrifugation, at 4°C for 15 minutes; and their protein concentrations were measured by BCA Protein Assay (Thermo Scientific). Equal amounts of total protein were loaded, resolved by SDS/PAGE using precast Novex gradient 4% to 20% (wt/vol) Tris–Glycine gels (Life Technologies), and electro-transferred to PVDF membranes. Such membranes were then blocked with Intercept blocking buffer (LI-COR Biosciences) and incubated with primary antibodies (1:200; Santa Cruz Biotechnology) and β-actin (1:5000; Cell Signaling Technology). After overnight incubation at 4°C with primary antibodies, secondary fluorophore-conjugated antibodies followed by imaging with LI-COR Odyssey CLx were used to measure fluorescence intensities and quantify them with Image Studio (LI-COR Biosciences). Densitometry measurements were expressed in relative fluorescence units (RFUs) normalized to β-actin.

### Quantitative PCR Analysis

RNA extraction was performed using RNAeasy kit (Qiagen) and measured using NanoDrop and standard spectrophotometric parameters. We retrotranscripted 2 µg of RNA from each sample to cDNA using High-Capacity cDNA Reverse Transcription Kit (Applied Biosystems). We used 2 µL of cDNA samples as a template for amplification reactions carried out with the TaqMan Fast Advanced Master Mix, TaqMan assay FAM for genes of interest, and *Hprt* TaqMan assay VIC (reference gene) in a QuantStudio 3 Real-Time PCR System (all from Applied Biosystems), and relative 2^−ΔΔ^*^Ct^* qPCR data analysis was performed with QuantStudio Design and Analysis software version 1.4.3 (Applied Biosystems). Expression of target genes was normalized to that of *Hprt* and presented as fold-change over the control.

### Seahorse Assay and Retinal Explant Harvest

Retinal explants were harvested from A/J and C57Bl/6 mouse strains following the protocol described by Jiang et al.^16^ Briefly, mice were enucleated, and their retinas were dissected away from the sclera/choroid/retinal pigment epithelium (RPE). Using a biopsy puncher, 1-mm diameter retinal punches were created along the periphery of the retina. These punches were placed on Seahorse Islet Capture Meshes and stuck on using Cell-Tak. Punches were run through the Seahorse XFe24 Extracellular Flux Analyzer following a protocol of five baseline measurements, Oligomycin injection, four measurements, rotenone and antimycin A injections, and four final measurements.

### Resipher Analysis

Choroidal sprouts were harvested and plated as previously described above. Sprouts were grown in a 96-well Falcon clear bottom plate and incubated with endothelial cell culture media for 7 days. Resipher assay (Lucid Scientific) was used to measure the real-time oxygen consumption of the choroidal sprouts over 48 hours. The oxygen consumption rates were averaged over the last 6 hours for improved stability of measurements and normalized to cell number using the Cell Viability Assay Kit (Abcam).

### Isotopic Metabolomics

Cells were recovered from cryopreservation and seeded in six-well culture plates. Upon reaching 90% confluence, media was replaced with media containing U^13^C_6_ Glucose at equal concentration. Cells were incubated for 24 hours to establish an isotopic steady state. Cells were washed with 0.9% NaCl, quenched in –80°C methanol, and evaporated to dryness under vacuum. One mL of ice-cold water was added to each well and cells were scraped on an ice block. The suspension was transferred to 1.5-mL tubes and dried under vacuum. The samples were then extracted in 400 µL CHCl_3_, 400 µL MeOH, and 400 µL H_2_O. The upper phases were dried under vacuum and derivatized in MTBSTFA at 70°C for 1 hour. Samples were packed in vials for gas chromatography/mass spectrometry (GC/MS) analysis. The Agilent 5977b mass spectrometer was manually tuned to achieve a PW50 of less than 0.5 AMU for each tuning ion. Standards of target metabolites were derivatized in parallel and used to determine the optimal SIM ions to the nearest 0.1 AMU. Isocor was used to correct mass spectra for natural isotopic abundance and to calculate mean enrichment.^20^ Mean enrichments and mass isotopomer distributions (MIDs) were compared using unpaired Welch's *t*-tests.

## Results

We first confirmed that the A/J mouse had the same mitochondrial mutation in the DHU loop of the transfer RNA for Arginine gene (mt-A; Fig. 1A) as albino BALB/c and FVB mice strains. The specific mitochondrial mutation for mt-A has nine Adenine residues instead of eight in the DHU loop (Figs. 1B, 1C).

**Figure 1.**
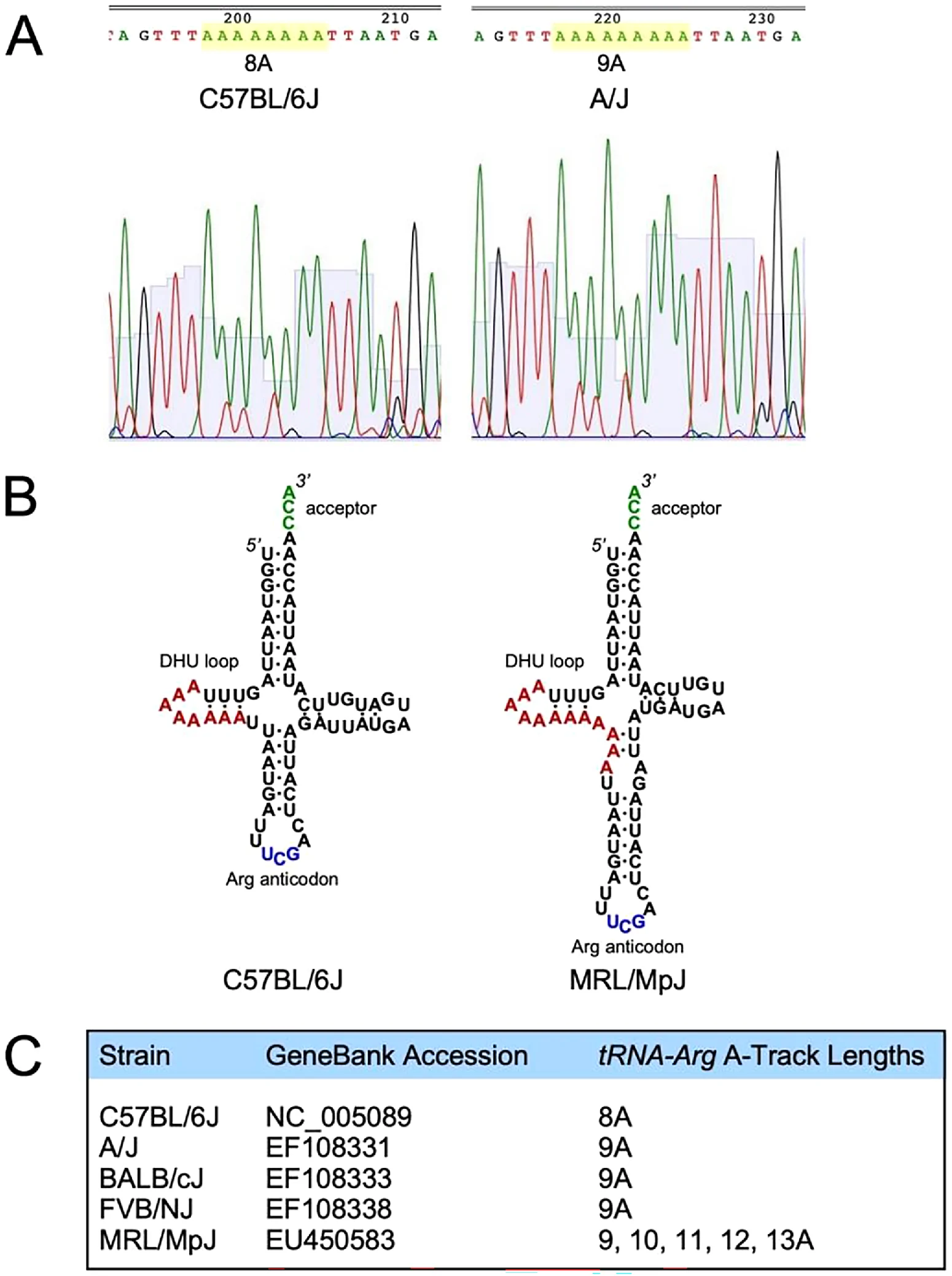
**Mitochondrial transfer RNA mutation.** (**A**) The A/J mouse harbors an arginine mitochondrial transfer RNA (mt-A) mutation. (**B**) The mutation adds an adenine to the DHU loop. (**C**) This mutation is present in various mouse strains resistant to OIR and is a marker of mitochondrial lineage.

We next tested the A/J mouse in the OIR model. The albino A/J mouse strain was highly protected from OIR compared with C57Bl/6 mice, having a 5-fold reduction in the ratio of vaso-obliteration (*P* = 1*10^−5^) and a 20-fold reduction in neovascularization (*P* = 0.002). Figure 2A compares C57Bl/6 to A/J, demonstrating less oxygen induced vaso-obliteration/neovascularization in the A/J mouse despite similar rates of revascularization (Fig. 2B). By mapping crosses between A/J and C57Bl/6 mice, we found a maternal pattern of inheritance for protection in the F1 generation that suggests the involvement of the mitochondrial genome (Figs. 2C, 2D). We next made certain that imprinting had no effect of OIR severity by cross-fostering pups from each lineage (Figs. 2E, 2F). The BALB/c mouse strain was also found to have increased protection from OIR from having a BALB/c maternal parentage compared to BALB/C paternal parentage, although this protection was less than F1 A/J × C57Bl/6 mice with A/J mothers (Figs. 2G, 2H). F1 phenotypes were equal in male and female mice, ruling out X-linked inheritance. Raw flatmount photos are in Supplementary Figure S1.

**Figure 2.**
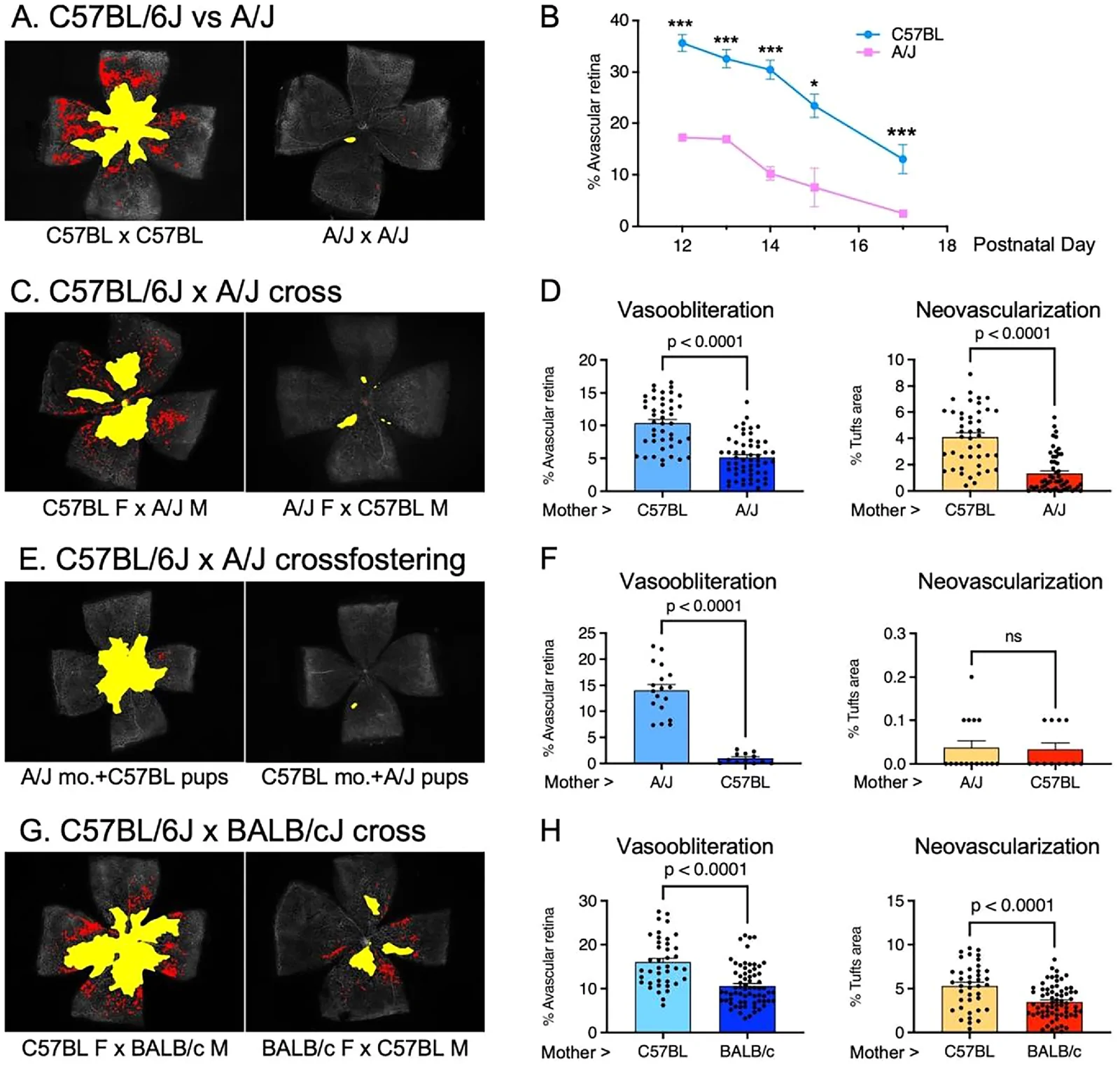
**Resistance to OIR follows vertical transmission from the mother.** (**A**) A/J mice are significantly protected from OIR. (**B**) Vaso-obliteration in A/J mice is less at P12 compared with C57Bl/6 mice although the rate of revascularization is the same. (**C**) F1 generations are protected only if the mother is from A/J lineage, quantified in **D**. (**E**) Imprinting is not a mechanism of protection, quantified in **F**. (**G**) BALB/c mice protection also follow vertical transmission, although not as strongly as A/J, quantified in (**H**).

Understanding that vertical transmission implies a mitochondrial basis for disease severity, we compared oxygen consumption (OCR) and extracellular acidification (ECAR), surrogate measurements for levels of oxidative phosphorylation and glycolysis, respectively, between mouse strains. Figure 3A compares oxygen consumption of C57Bl/6 versus A/J retinal explants treated with oligomycin and rotenone to inhibit Complex V/I to stop mitochondrial respiration. A/J mice had less basal oxygen consumption (*P* = 0.018) and proton leak (*P* < 0.001), whereas ATP-linked respiration was not significantly different (see Figs. 3A, 3B).

**Figure 3.**
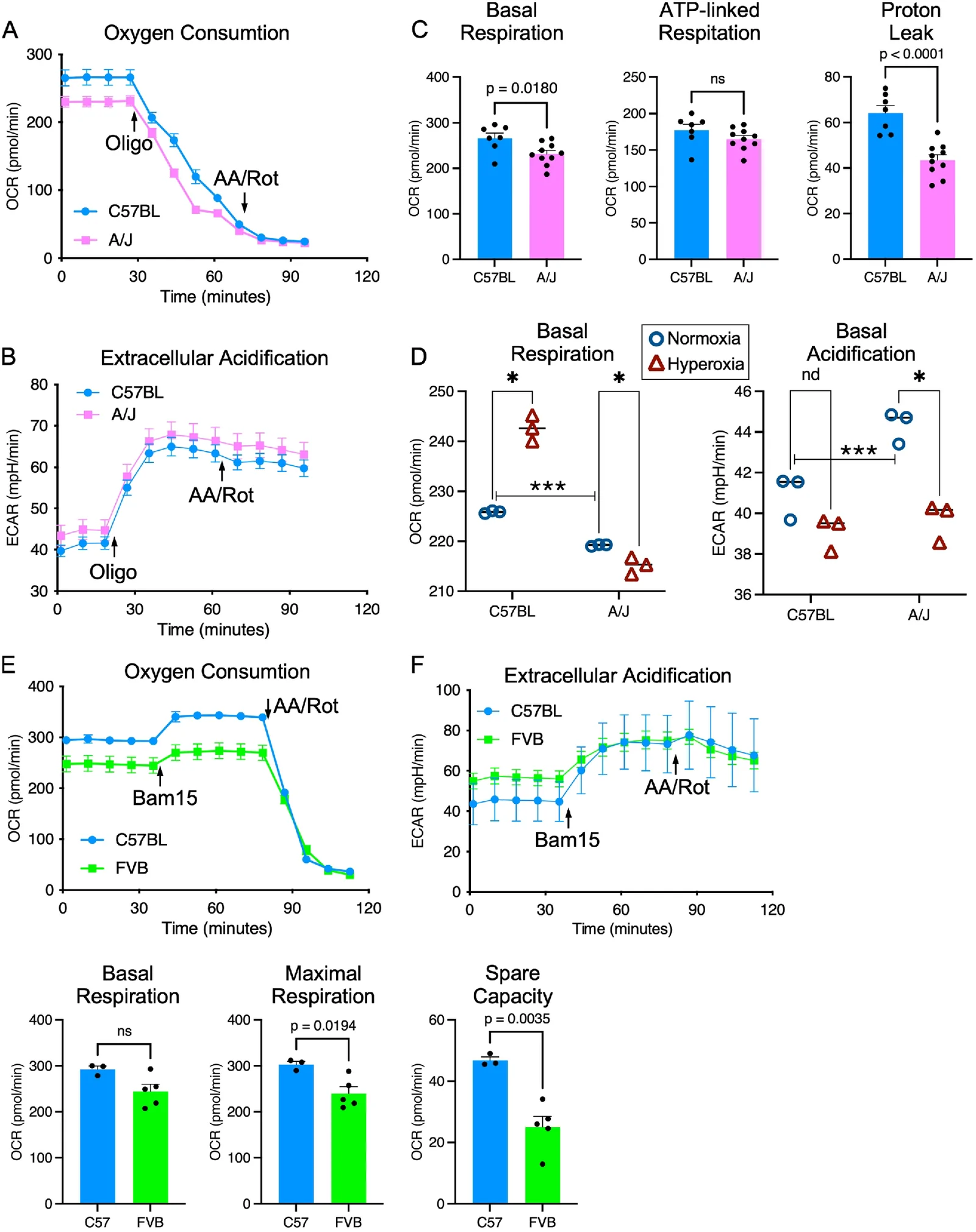
**Oxygen consumption and extracellular acidification of retinal explants.** (**A**) Comparison of OCAR between A/J and C57Bl/6 mice demonstrates less ATP generating oxygen consumption by A/J and (**B**). increased glycolytic extracellular acidification by A/J in hyperoxia. (**C**) Baseline OCAR is less in A/J retinal explants (**D**). A/J demonstrates decreased, and Blk6 increased expression of the critical Cox4i1 and Cox 4i2 subunits of cytochrome C oxidase in both normoxia and hyperoxia. (**E**) Like A/J, FVB mice have decreased basal OCAR and (**F**) preserved glycolytic function.

The same inhibitors were used to isolate glycolysis to measure ECAR. A/J had a lower basal glycolytic rate than C57Bl/6 but similar overall rates of ATP linked glycolysis (Fig. 3C). Figure 3D compares basal OCR, less in A/J mice, and ECAR, increased in A/J.

Additional seahorse experiments with the FVB mice also showed the same lower basal rate of OCR (Fig. 3E; *P* = 0.005) and ATP-linked respiration (*P* = 0.05). Like A/J, basal glycolysis measured by ECAR is greater in FVB mice (Fig. 3F).

We tested whether decreased respiration correlated with decreased Complex I to V protein concentration. Densitometry of Western blotting demonstrate that AJ mice have decreased mitochondrial Complex I, II, III, and V protein levels in the retina (Fig. 4A, Western blots in Supplementary Fig. S2). Survey of key genomic and mitochondrial gene expression was notable for equivalent retinal expression patterns, with the striking exception of heme oxygenase 1 (*Hmox1*) which was increased 4-fold over levels of C57Bl/6 (Fig. 4B; *P* = 0.002). We observed decreased mRNA in both C57Bl/6 and A/J mice for the critical HIF-dependent *Cox4i2* subunit of cytochrome c oxidase (Complex IV, *P* < 0.000001) during hyperoxia; this was opposite to C57Bl/6 who demonstrated elevated expression of the *Cox4i1* subunit. These findings of C57Bl/6 increased expression of the *Cox4i1* subunit in hyperoxia correlates to the higher OCR in this strain. Schema in Figure 4E demonstrates HIF-independent *Cox4i1* active even in hyperoxia in susceptible C57Bl/6 retinal mitochondria. Prevailing *Cox4i1* subunit over the HIF-dependent *Cox4i2* is documented to give rise to the production of reactive oxygen species (ROS) by mitochondrial electron transport chain ETC^21^). We further tested “resistance” against hyperoxia in A/J choroidal explants which demonstrated increased growth even in hyperoxia compared with C57Bl/6 choroidal explants (see Fig. 4E, explants shown in Supplementary Fig. S3).

**Figure 4.**
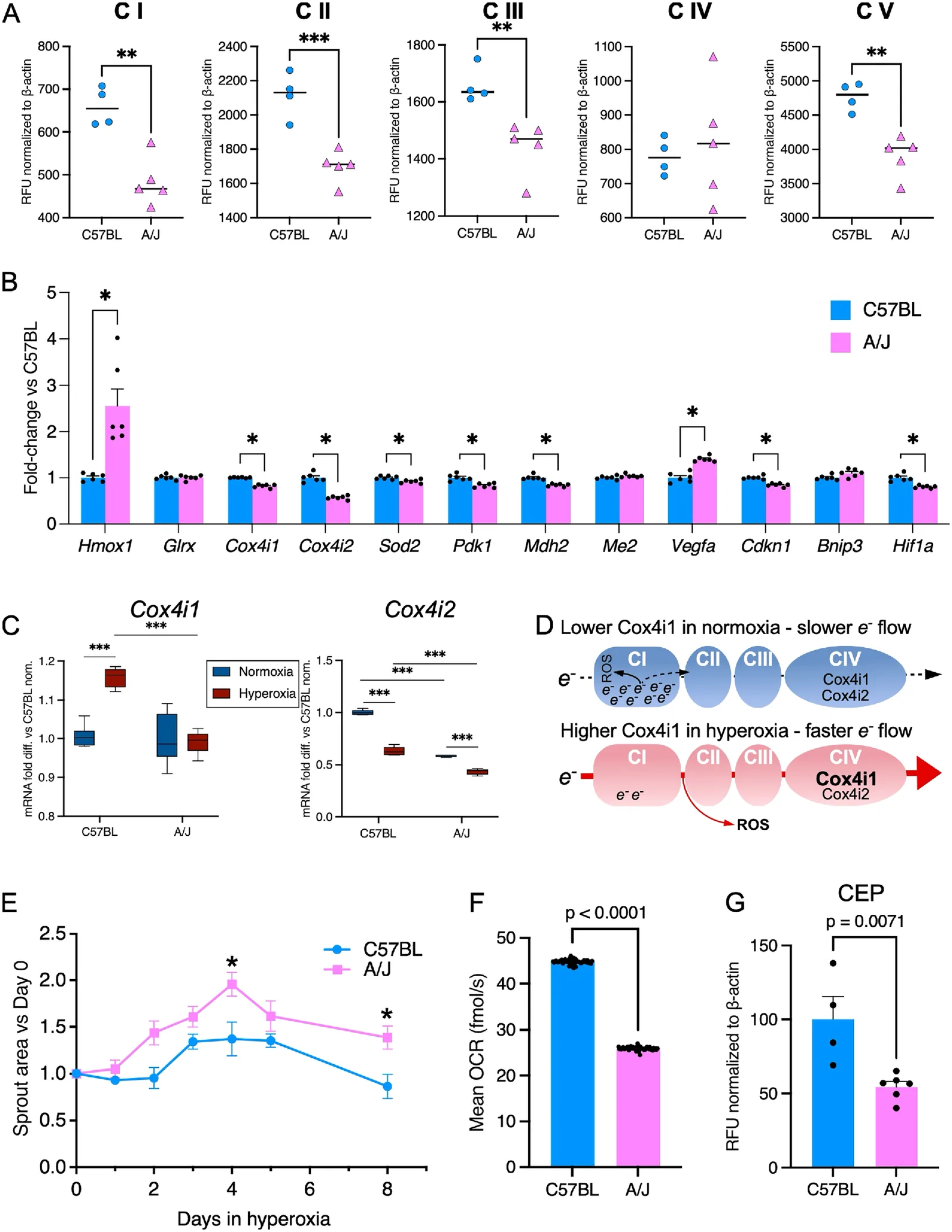
**Survey of strain-specific metabolic enzymes.** (**A**) Densitometry of Western blot (Supplementary Fig. S1) demonstrates reduced protein concentrations of Complex I, II, II, and V. (**B**) The qPCR of key metabolic nodes reveals increased expression of *Hmox1* in A/J retina. (**C**) The Cox4 subunits ½ are critical to cytochrome c oxidase. The HIF-dependent *Cox4i2* is decreased in hyperoxia, but the *Cox4i1* subunit is overexpressed in C57Bl/6 retina alone. (**D**) Schema showing ROS-generating *Cox4i1* is elevated in hyperoxia in the susceptible strain. (**E**) Choroidal endothelium of A/J grows faster in hyperoxia compared to C57Bl/6. (**F**) A/J endothelium grow faster but consume less oxygen. (**G**) A/J retina harbors less oxidative damage shown by content of carboxyethyl pyrrole (CEP) adducts.

In addition, this increased growth was accompanied by reduced OCR in A/J mice compared to C57Bl/6 mice (Fig. 4F, *P* < 0.0001). In the retina, we found decreased carboxyethyl pyrrole (CEP) modifications in retina, an oxidation induced Schiff base adduction (Fig. 4G, Supplementary Fig. S4). We observed a 2-fold reduction in the CEP oxidative stress marker of A/J mice retinas that underwent OIR (*P* = 0.007) compared with C57Bl/6 mice.

Given the reliance on glycolysis, we next tested differences in glucose oxidation between mouse aortic endothelial cells (MAECs) from each strain using fully labeled glucose. At equilibrium, A/J aortic endothelial cells demonstrated an increase in labeled citrate (Figs. 5A, 5B) confirming entry of glycolytic carbon into the tricarboxylic acid cycle (TCAC) compared to C57Bl/6, but without relative increases in succinate (Fig. 5C) or fumarate (Fig. 5D), especially the M2/4 isotopomers. Instead, A/J MAECs synthesized 15% more glutamine (Gln) than C57Bl/6 endothelium from glycolytic carbon (see Figs. 5A, 5E).

**Figure 5.**
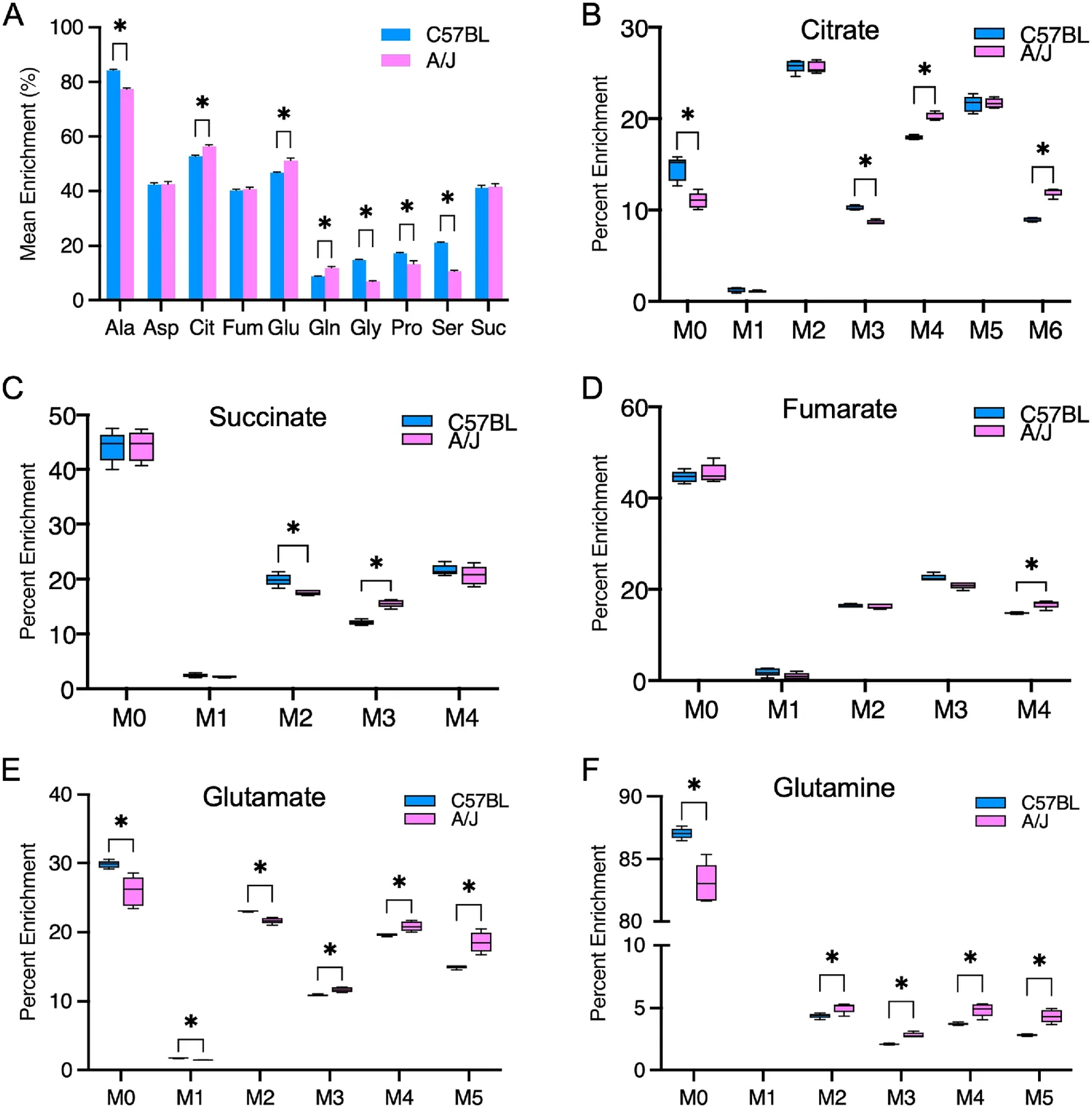
**A/J MAECs (mouse aortic endothelial cells) synthesize more Gln from glycolytic carbon than C57/Bl6 endothelia.** (**A**) Mean enrichment shows increases in citrate, glutamate, and Gln in endothelial cells from A/J compared to C57/Bl6. (**B**) M4 Citrate is slightly elevated. (**C**) Succinate M2/4 isotopomers are comparatively decreased in A/J endothelia (**D**) as is fumarate. (**E**) M3/4/5 isotopomers of glutamate are increased. (**F**) Gln levels derived from glucose are similarly increased in A/J endothelium.

Puzzled by the ability of choroidal explants to grow faster even in hyperoxia despite reduced electron transport (less efficient energy generation), we sought to separate the hypothetical protective effects Gln/Hmox1 versus decreased ATP synthesis by disabling the electron transport in the susceptible C57Bl/6 strain to determine the effect of energy deprivation on OIR. We treated C57Bl/6 pups with DNP, a potent mitochondrial uncoupler agent that short circuits the proton gradient across the mitochondrial membrane to disconnect electron transport from ATP synthesis. A two-fold reduction in avascular retina is seen in animals treated with DNP (Figs. 6A, 6B, Supplementary Fig. S5). This provides a working hypothesis illustrated in Figures 6C and 6D suggesting a mitochondrial-based protection centered on reduced electron transport and stable aerobic glycolysis, also known as the Warburg effect.

**Figure 6.**
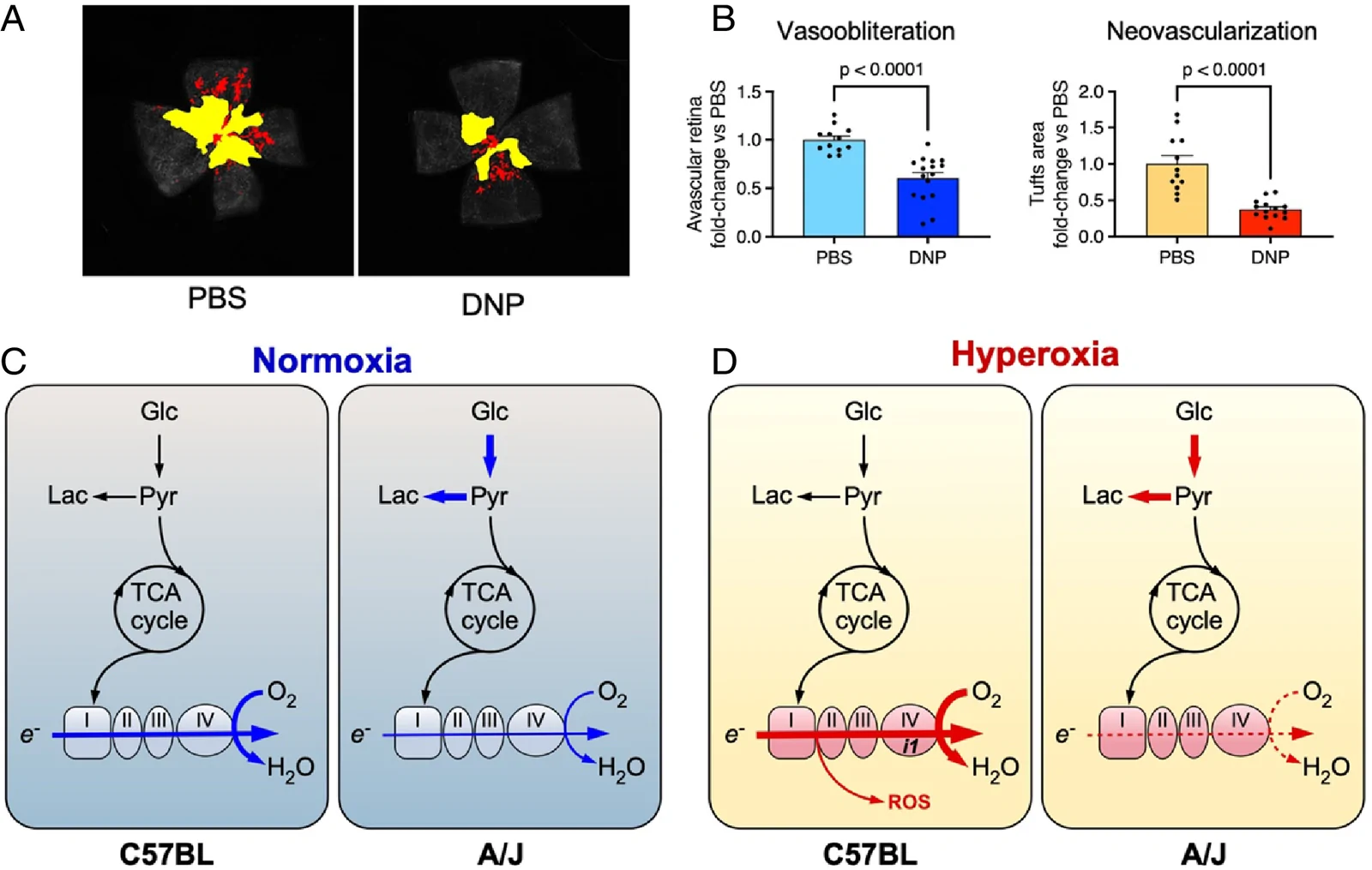
**Mitochondrial uncoupler and OIR.** (**A**) Flatmounts and (**B**) quantification of avascularity and neovascularization demonstrates reduction of OIR vascular pathology. (**C**) In normoxia, A/J (*right panel* in **C**) is more glycolytic than C57Bl/6 (*left panel* in **C**) which resembles the Warburg effect. (**D**) In hyperoxia, A/J respiration is even lower. Hyperoxia stimulates oxygen consumption in C57Bl/6 retina and upregulates *Cox4i1*, an ROS-generating subunit (*left panel* in **D**). Disabling ETC by DNP simulates A/J metabolism (*left panel* in **D**) and protects mouse retinal vasculature from hyperoxia by decreasing reactive oxygen species (ROS).

## Conclusions

The A/J mouse provides an opportunity to define the relationship between mitochondria and susceptibility/protection against OIR. The maternal vertical transmission we observed in the A/J mouse might reflect strain-specific mitochondrial genetics, as strains from similar genetic backgrounds harbor similar mitochondrial polymorphisms. We confirmed that other albino mice strains including A/J had a mitochondrial tRNA polymorphism; notably mt-tRNA mutations have been shown to be pathogenic through interference with mitochondrial translation and oxidative phosphorylation. We know the protective gene(s) is not X-linked as F1 male and female mice had similar phenotypes.

A constant finding in this investigation was the reduced oxygen consumption in the A/J and FVB strain. Reduced oxidative phosphorylation may be driven by the difference in subunits of Complex IV in hyperoxia.^22^ Expression of *Cox4i2* was downregulated by hyperoxia in both strains. Because it is HIF-dependent, it is expected to demonstrate decreased expression in hyperoxia. In contrast, subunit *Cox4i1* is HIF independent.^23^ We observed increased expression of this critical subunit *Cox4i1* in the retina of susceptible C57Bl/6 mice in hyperoxia. In fact, we observed a two-fold increase in protection against OIR by uncoupling electron transport by DNP suggesting that disabling electron transport might create protection, implying that glycolysis is protective and that OIR is not an energy deficit. Previous reports of metabolic differences between A/J and C57Bl/6 mice found that A/J mice fed a high-fat diet had less hepatic fat deposition compared with similarly fed C57Bl/6 mice through a decrease in ATP generating OCR.^24^ Although uncoupling should increase not decrease OCR, our experimental conditions of hyperoxia are different than a high-fat diet and supports the hypothesis that decreasing ATP generated by electron transport might be protective against OIR.

We also observed that there was avascular retina present at the close of phase 1 (P12) in both the A/J and C57Bl/6 strains, but much less in the A/J. This also might lead to faster revascularization in phase 2. This is supported by the observation that choroidal endothelium from the A/J mouse grew better in hyperoxia than C57Bl/6 yet consumed less oxygen during the growth phase.

In the manuscript by Yagi et al., the authors found that decreased oxidative phosphorylation during hypoxia was associated with pathologic angiogenesis. Our investigation may demonstrate that the benefit of decreasing respiration may be phase 1 or phase 2 sensitive, as we found a decrease in respiration in hyperoxia to be associated with protection. Moreover, A/J endothelium used the TCAC to synthesize glutamine from glucose during hyperoxia.

The decrease in respiration did not prevent glycolytic carbon from entering the TCAC, but rather A/J used the TCAC to synthesize Gln. Gln is necessary for increasing endothelial cell biomass^25^^–^^27^ in phase 2 revascularization. Astrocytes oxidize more Gln in hyperoxia; this might deprive endothelial cells of Gln creating retinovascular growth attenuation.^18^^,^^25^ Synthesis of Gln by A/J endothelium might therefore compensate for Gln consumed by astrocytes and could explain more rapid growth of A/J endothelium than C57Bl/6.

The four-fold increase in *Hmox1* expression is also relevant to endothelial growth, as microglial *Hmox1* has been linked to retinal endothelial proliferation via STAT-3.^28^ Heme oxygenases catalyze the rate-limiting step in heme degradation, resulting in the formation of iron, carbon monoxide, and biliverdin, which are subsequently converted to bilirubin by biliverdin reductase.^29^ Gln exerts potent antioxidant and anti-inflammatory effects in the circulation by inducing the expression of *Hmox1*, heat shock proteins, and Gln.^30^ Moreover, both Gln and *Hmox1* are reported to act synergistically to protect blood vessels during ischemia mediated injury, shock, and acute kidney injury, acting through p38 MAPk.^31^^–^^33^ Ammonia can also induce *Hmox1* expression; ammonia has been reported to be associated with severe prematurity and severe ROP.^34^^,^^35^

In conclusion, the A/J mouse provides a platform to test how metabolism plays a role in OIR. The A/J mouse demonstrates maternal vertical inheritance pattern of protection through maternal lineage, implicating mitochondrial influence. Our finding that decreased OCR, lower protein levels in the respiratory chain, and particularly oxygen insensitivity of *Cox4i1*, might underlie increased cytoprotection, at least in the response to hyperoxia, and implies that glycolysis, which was increased, is protective. Furthermore, we find that the A/J mice utilize glucose differently, using cataplerotic glycolytic carbon to synthesize more Gln than in OIR-susceptible C57Bl/6 mice, upregulating *Hmox1*. The inherent protection of A/J mice against OIR follows diverse metabolic differences between susceptible and protected strains. Our results suggest that metabolic protection in phase 1 hyperoxia involves decreasing oxidative phosphorylation with a reliance on aerobic glycolysis, reducing ROS formation. When glucose does enter the TCAC in hyperoxic A/J mice, glycolytic carbon becomes cataplerotic to form glutamine.

## Supplementary Material

Supplement 1

Supplement 2

Supplement 3

Supplement 4

Supplement 5
