# Supplementary material for: Maternal Vertical Transmission Provides a Mitochondrial Basis for ROP Susceptibility
Source: Invest Ophthalmol Vis Sci. 2026 Oct 30;67(12):78. doi: 10.1167/iovs.67.12.78 (PMC13722966; doi:10.1167/iovs.67.12.78)

Figure S1

Supplemental Figure 1

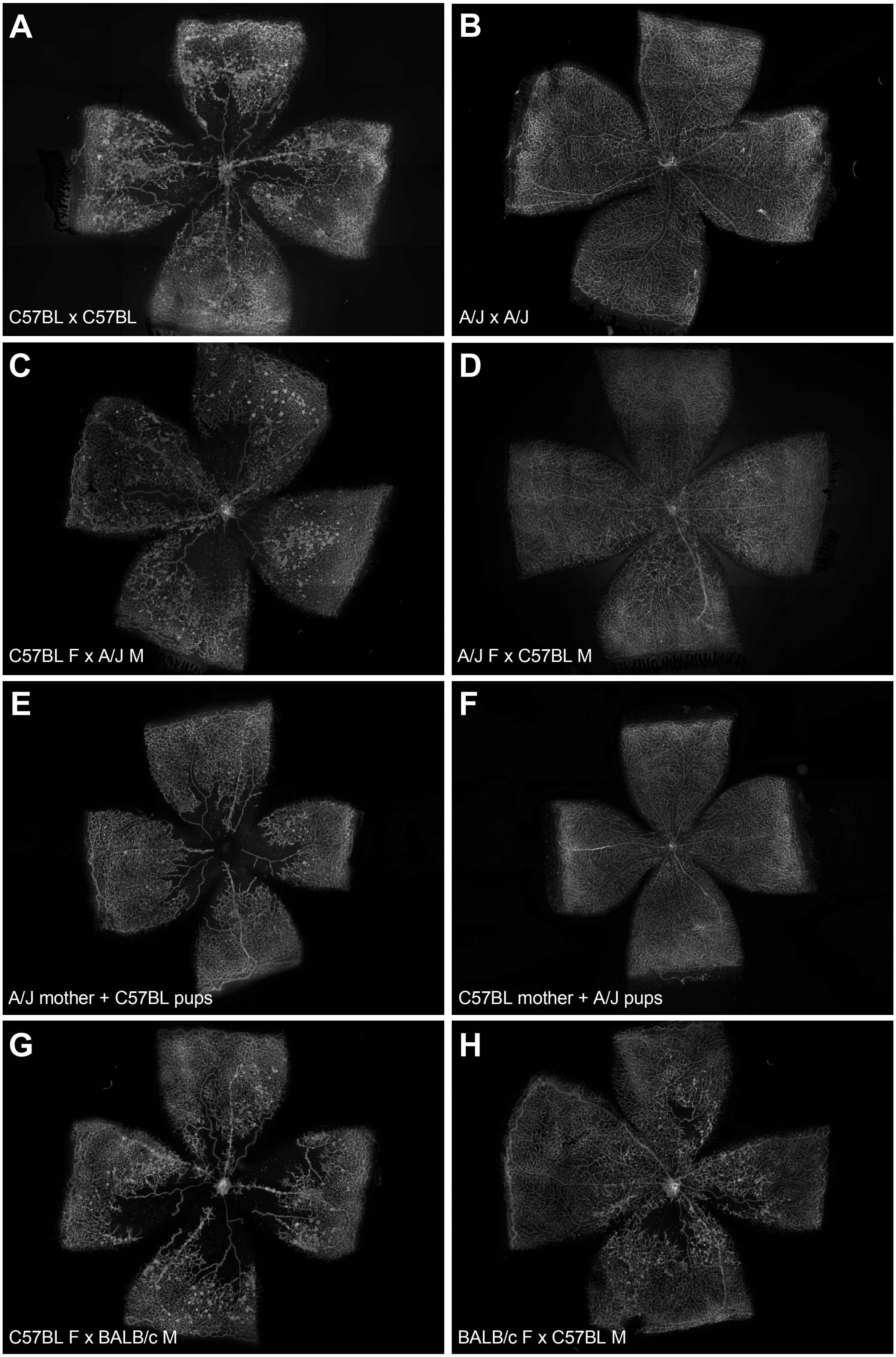

Supplement: Supplementary file 1 [file iovs-67-12-78_s001.pdf]
