# Supplementary material for: Maternal Vertical Transmission Provides a Mitochondrial Basis for ROP Susceptibility
Source: Invest Ophthalmol Vis Sci. 2026 Oct 30;67(12):78. doi: 10.1167/iovs.67.12.78 (PMC13722966; doi:10.1167/iovs.67.12.78)

Figure S3

Figure S3

Supplemental Figure 3

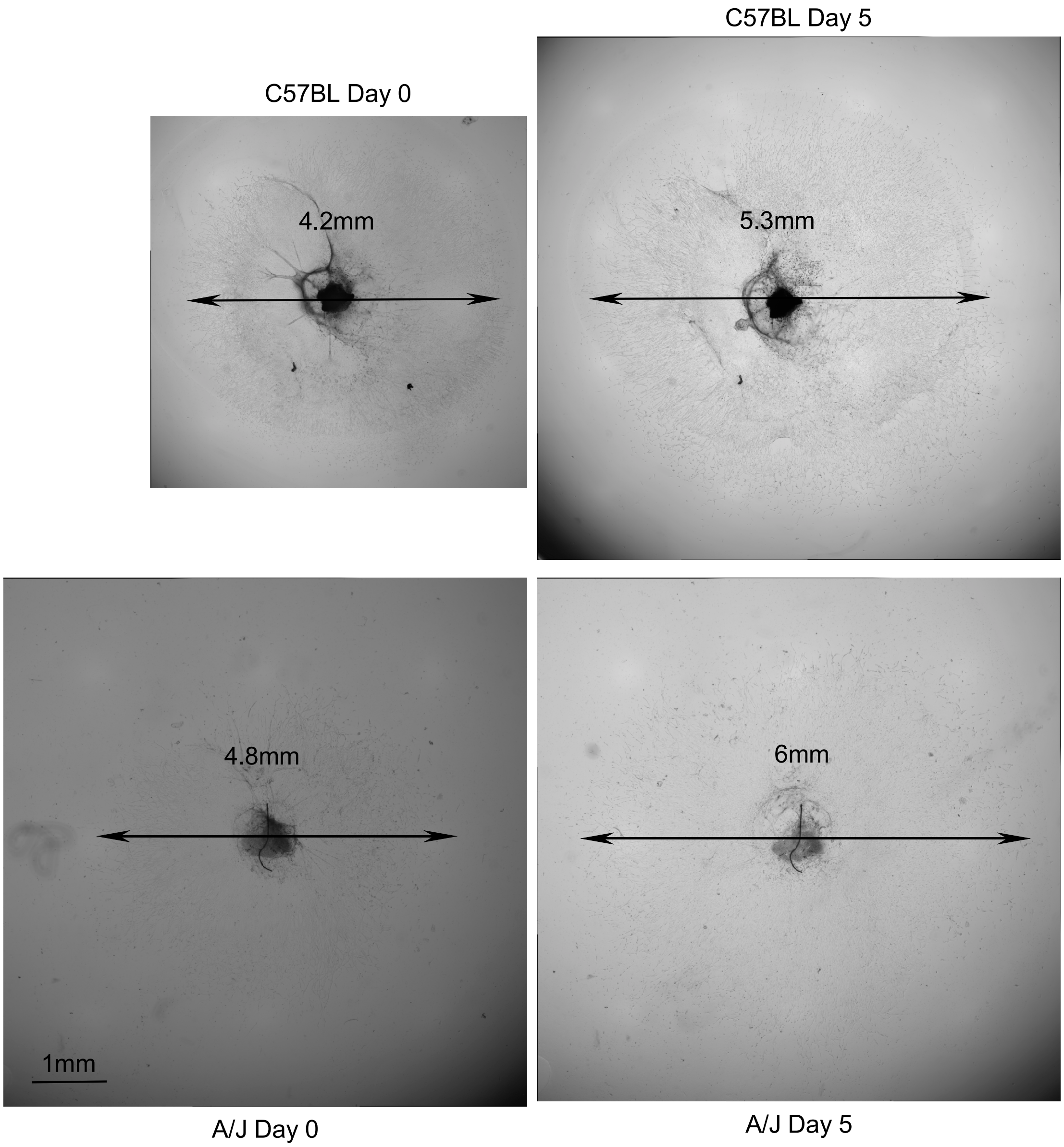

Supplement: Supplementary file 3 [file iovs-67-12-78_s003.pdf]
